# Connexin hemichannels as candidate targets for cardioprotective and anti-arrhythmic treatments

**DOI:** 10.1172/JCI168117

**Published:** 2023-03-15

**Authors:** Luc Leybaert, Maarten A.J. De Smet, Alessio Lissoni, Rosalie Allewaert, H. Llewelyn Roderick, Geert Bultynck, Mario Delmar, Karin R. Sipido, Katja Witschas

**Affiliations:** 1 Physiology Group, Department of Basic and Applied Medical Sciences, Ghent University, Ghent, Belgium.; 2 Laboratory of Experimental Cardiology, Department of Cardiovascular Sciences, and; 3 Laboratory of Molecular and Cellular Signaling, Department of Cellular and Molecular Medicine, KU Leuven, Leuven, Belgium.; 4 Leon H. Charney Division of Cardiology, School of Medicine, New York University, New York, USA.

## Abstract

Connexins are crucial cardiac proteins that form hemichannels and gap junctions. Gap junctions are responsible for the propagation of electrical and chemical signals between myocardial cells and cells of the specialized conduction system in order to synchronize the cardiac cycle and steer cardiac pump function. Gap junctions are normally open, while hemichannels are closed, but pathological circumstances may close gap junctions and open hemichannels, thereby perturbing cardiac function and homeostasis. Current evidence demonstrates an emerging role of hemichannels in myocardial ischemia and arrhythmia, and tools are now available to selectively inhibit hemichannels without inhibiting gap junctions as well as to stimulate hemichannel incorporation into gap junctions. We review available experimental evidence for hemichannel contributions to cellular pro-arrhythmic events in ventricular and atrial cardiomyocytes, and link these to insights at the level of molecular control of connexin-43–based hemichannel opening. We conclude that a double-edged approach of both preventing hemichannel opening and preserving gap junctional function will be key for further research and development of new connexin-based experimental approaches for treating heart disease.

## Introduction

The coordinated functioning of the heart as a pump heavily relies on communication between more than 3 billion cardiomyocytes via connexin-based gap junctions (GJs). GJs connecting cardiomyocytes and cells in conduction tissues distribute electrical impulses over this cellular syncytial network, acting to steer and synchronize the contraction-relaxation sequence of atria and ventricles during each pump cycle. GJs are a special kind of channel, as they are composed of two hemichannels (HCs) originating from distinct juxtaposed cells sharing the junctional channel. Like their HC counterparts, GJs pass ions, metabolites, and signaling molecules with a molecular weight (MW) below about 1.5 kDa, e.g., Na^+^, K^+^, Ca^2+^, ATP, cAMP, inositol 1,4,5-trisphosphate, and many others (1–3).

The importance of GJs becomes clear from the huge turnover rate of its connexin building blocks, characterized by a half-life of approximately 1–5 hours for the 43-kDa connexin-43 (Cx43), a major connexin in the heart (4, 5). Such a high turnover rate allows continuous remodeling of GJs to adapt to varying degrees of cardiac pump load. This is associated with a continuous Cx43 stream through the endoplasmic reticulum/Golgi network, where Cx43 oligomerizes (6), followed by its arrival in the plasma membrane assembled as hexameric HCs. From there, HCs migrate laterally in the sarcolemmal lipid bilayer toward a zone called the perinexus where they coalesce with the edges of existing GJs, known as the nexus zone (4, 7). HCs subsequently dock head to head with their counterparts on neighboring cells, forming GJ channels that combine into channel arrays organized as junctional plaques. The connexin’s life as a GJ ends with plaque-associated GJs being internalized as “annular GJs” or connexosomes, which are further degraded via endolysosomal or autophagosomal pathways (refs. 4, 8–11; and reviewed in ref. 12).

The high Cx43 turnover rate also implies a continuous process of HC delivery to the sarcolemma. Before their incorporation into GJs, HCs are closed to prevent membrane leakage of intracellular and extracellular factors through these highly conductive channels (~220 pS electrical conductivity per Cx43 HC; compare with ~5 pS for L-type Ca^2+^ channels). While open GJs close under certain conditions, such as ischemia, HCs react oppositely and open under ischemic or inflammatory conditions (13–16). The functional consequences of HC opening have been difficult to investigate under ex vivo or in vivo conditions, mainly because connexin channel inhibitors used for this purpose affect both GJs and HCs. Since the finding that certain Cx43 mutants distinctly affect HC versus GJ function (17, 18) and the introduction of peptides such as Gap19 that inhibit Cx43 HCs without inhibiting GJs (19), more detailed and conclusive studies have been possible. Here, we discuss how inhibition of HCs can be used to protect against experimental ischemic cardiac injury and review recent experimental evidence on their possible contribution to arrhythmogenic processes. Considerations on mitochondrial Cx43 HCs are not covered here, as these have been recently reviewed elsewhere (20). For background information on the role of GJ channels and HCs in cardiac ischemia and arrhythmia, we refer the reader to comprehensive reviews in refs. 12, 21, and 22.

## Preserving GJs and inhibiting HCs in cardiac disease

**Figure 1:**
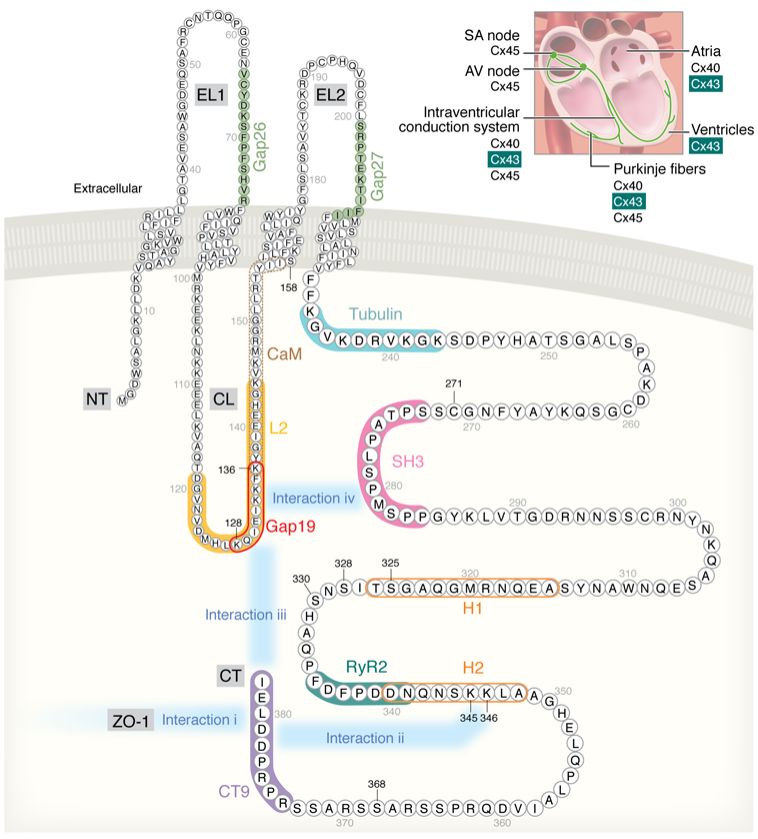
Connexins in the heart and Cx43 topology with indication of crucial sites and interactions that are discussed in this Review. The upper right image illustrates expression of Cx40, Cx43, and Cx45 in heart muscle, conduction tissues, and nodes. Cx43 is present in most of the tissues, except for the sinoatrial and atrioventricular nodes. Cx40 prevails in atrial cardiomyocytes and in the His-Purkinje system (23, 25, 152, 153), while Cx45 is primarily expressed in the conduction system (23). The Cx43 topology scheme indicates various regions and sequences that are discussed in this Review. Gap19, L2, Gap26, Gap27, and CT9 are sequences that have been used as mimetic peptides affecting Cx43 channel function as well as Cx43 interaction within or outside the protein. The sequences marked “Tubulin” and “RyR2” (ryanodine receptor-2) indicate interaction sites with these respective proteins. “CaM” indicates a calmodulin interaction site. “SH3” denotes an Src homology binding domain. H1 (315 to 326) and H2 (340 to 348) are α-helical structures. Ser325, Ser328, Ser330, and Ser368 are phosphorylation sites, while Cys271 is a nitrosylation site, which are discussed in the main text. NT, N-terminal end; CT, C-terminal end; EL1 and EL2, extracellular loops 1 and 2; CL, cytoplasmic loop; ZO-1, zonula occludens 1.

Cardiomyocytes and cells of the conduction system in the normal human adult heart mainly express Cx40, Cx43, and Cx45 (23–25). Cx43, a ubiquitously expressed connexin encoded by the *GJA1* gene, is the main connexin in ventricular cardiomyocytes, and in the His-Purkinje system, which facilitates rapid electrical conduction in heart ventricles (23–25) (Figure 1).

The roles of GJs formed by atrial Cx40/Cx43 and ventricular Cx43 have been extensively investigated in cellular and animal models of cardiac ischemia/reperfusion (12, 26). GJs close under ischemic conditions as a consequence of cytosolic modulation by intracellular acidification, increased cytoplasmic Ca^2+^ concentration ([Ca^2+^]_i_), and connexin dephosphorylation (27–29), thereby hampering impulse conduction over the specialized conduction tissues and myocardial tissue. However, cardiac impulse conduction is characterized by a large degree of “coupling reserve,” meaning that Cx43 protein levels must dip below approximately 20% before impulse conduction velocity decreases or the QRS interval, which partially reflects the fast upstroke action potential (AP) depolarization phase, becomes prolonged (30–33). Impulse conduction involves two components: voltage-gated Na^+^ channels propagating the depolarization wave over the sarcolemma and GJs passing the signal to neighboring cells (34). Moreover, Cx43 protein levels affect sarcolemmal Na^+^ channel expression at the intercalated disk (ID) (35), which introduces some nonlinearity in the relationship between Cx43 expression and impulse conduction. Moreover, coupling by so-called “ephaptic” mechanisms is also thought to contribute, entailing extracellular charge accumulation at the ID that is capacitively coupled to the adjacent cell interior, pushing it above threshold to promote AP propagation (reviewed in refs. 36, 37). Given the substantial coupling reserve and the many factors that influence cardiac AP propagation, it was somewhat surprising that enhancement of GJ coupling by preservation of Cx43 phosphorylation with ZP123 peptide, or its non-peptide version Gap134 (also known as danegaptide), limited infarct size, maintained conduction velocity, and prevented ventricular fibrillation in acute coronary ischemia studied in various animal models (38–44). Unfortunately, no such benefit was observed in a human clinical study in which effects of danegaptide on infarct size or left ventricular ejection fraction were examined in myocardial infarction patients 3 months after thrombolysis treatment (45). Several reasons may underlie the lack of effect, including variability in patient pathology, incomplete mechanistic understanding of GJ enhancers, and the risky business of focusing on GJs as a single target. In fact, the effect of GJ enhancers in animal models of brain disease, which aim to preserve astrocytic syncytial connectivity, is also variable, as they reduce infarct size in stroke but enhance seizure activity in epilepsy (refs. 46, 47; and reviewed in ref. 48).

Around the turn of the century, evidence became available demonstrating HC opening in cardiomyocytes exposed to metabolic inhibition or ischemia/reperfusion (13, 14, 49), and showing protective effects of HC inhibition with Gap26 or Gap27 peptides against ischemic injury in isolated Langendorff-perfused rat hearts (50–52). Gap26/27 peptides, mimicking sequences on the extracellular loops of Cx43 (Figure 1), rapidly inhibit HCs and, with some delay, also inhibit GJs (ref. 53; and reviewed in ref. 22). The latter effect is obviously not acceptable for realistic treatment purposes. Further work with the more selective Cx43 HC peptide inhibitor Gap19, a nonapeptide sequence in the cytoplasmic loop (CL) (Figure 1), demonstrated protection of ventricular cardiomyocytes against metabolic inhibition–induced volume overload and cell death (19). Gap19 treatment also protected against cardiac ischemia/reperfusion injury in mice, reducing the infarct size by approximately one-fifth (19) (Table 1). Recent work with Gap19 in Langendorff-perfused rat hearts demonstrated approximately one-fourth infarct size reduction (54). For comparison, in a mouse permanent stroke model, treatment with TAT-Gap19 (containing the HIV-TAT cell-permeation sequence), aiming to target astrocytic Cx43 HCs, gave an approximately three-fourths reduction in brain infarct (55). It remains to be determined whether TAT-Gap19, which has a 5-fold better membrane permeability compared with Gap19 (19), could enhance its protective potential against cardiac ischemia/reperfusion injury.

αCT1 is another Cx43-based peptide that protects against ischemia/reperfusion injury in Langendorff-perfused mouse hearts (56). It is composed of the last 9 amino acids of the C-terminal (CT) end of Cx43 (Figure 1, CT9) coupled to an antennapedia cell-permeation sequence. Pre-ischemic administration of αCT1 demonstrated a strong improvement of left ventricular contractile function, and protection was even larger with CT9 peptide without antennapedia sequence, dubbed αCT11 (56) (Table 1). As CT9/αCT11 is not membrane permeable, it was hypothesized that the peptide entered the cells through open HCs.

Mechanistically, αCT1 prevented Cx43 remodeling known as “Cx43 lateralization,” i.e., increased Cx43 presence at lateral sides close to the ID, in a left ventricular cryoinjury model (57). It also reduced inducible arrhythmias and increased ventricular depolarization rate. The αCT1/αCT11 peptides are proposed to interfere with the interaction of the Cx43 CT (last 4 amino acids: DLEI sequence) with the PDZ2 binding domain of the zonula occludens scaffolding protein ZO-1 (58–60) (Figure 1, interaction 1). Consequently, Cx43 HCs lose their association with ZO-1, dock with their counterparts on the opposed membrane, and integrate as GJ channels at the edge of GJ plaques, a process that decreases HC presence in the perinexus and thus reduces Cx43 lateralization.

Besides interaction of the Cx43 CT end with the PDZ2 binding domain, the CT9 region of Cx43 also engages in molecular interactions with the CT-located α-helical H2 region (Figure 1, interaction 2). The αCT1/αCT11 peptides also interfere with this CT9-H2 interaction, thereby enhancing PKCε phosphorylation of Ser368 (Figure 1), which is crucial for their protective effects (56, 57). Phosphorylation of Ser368 is known to restrain the passage of high-MW substances through HCs (61–63), which may contribute to the protective potential of αCT1/αCT11. At the level of GJs, Ser368 phosphorylation enhances cell-cell coupling in response to ZP123 peptide (64), but decreased coupling has also been reported (65, 66).

## Molecular interactions controlling Cx43 channel function

Connexins located in the perinexus and nexus interact with multiple partners, including cytoskeletal molecules, mechanical junctions, and ion channel complexes (67), which affect their transport and organizational stability as well as channel functions. Most of these interactions, some of which have already been touched upon, involve the CT tail of Cx43 that forms a true hub, affecting not only connexin channel transport and function but also the activity of the interacting partner proteins. Below, we briefly highlight some of these interactions for their involvement in opposite effects on GJs versus HCs and their importance in HC-linked arrhythmogenic signaling.

### Cx43 tail-loop interactions govern HC opening.

**Figure 2:**
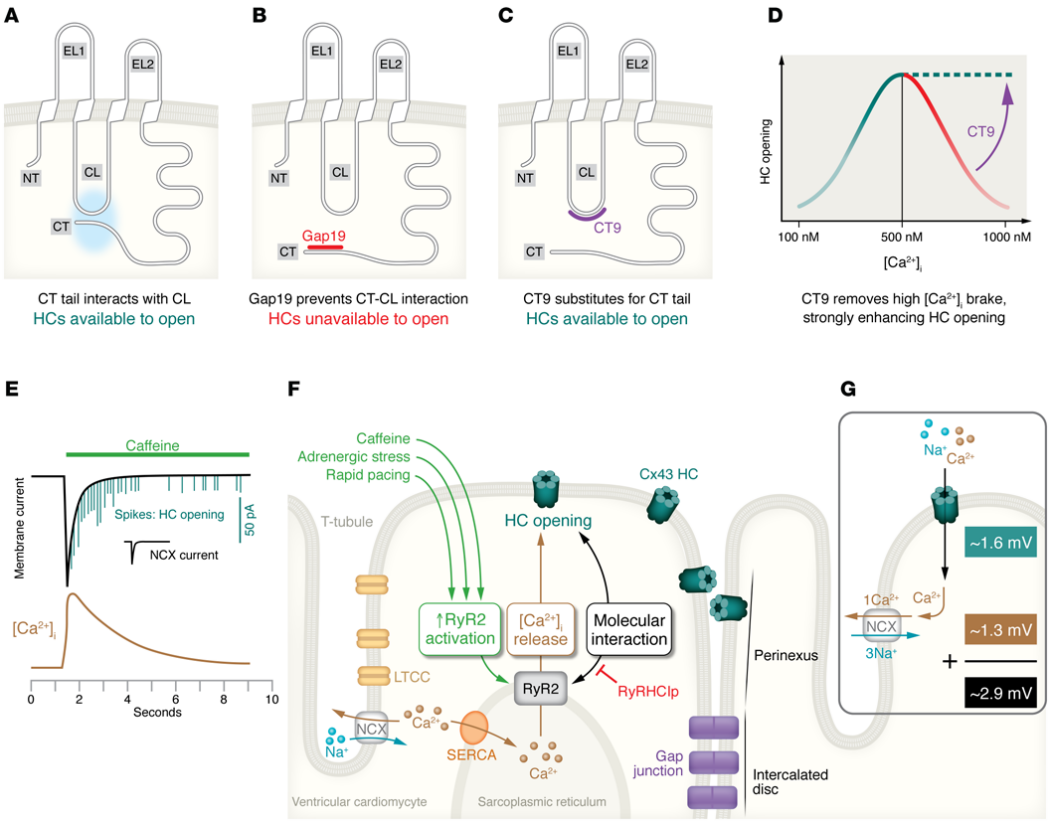
Cx43 HC activation in ventricular cardiomyocytes. (**A**–**C**) Cx43 HC states and the effect of Gap19 and CT9 peptides. (**A**) Interaction of the CT tail with the CL brings HCs into the “available to open” state. (**B**) Gap19 interaction with the CT prevents CT-CL interaction, making HCs unavailable for opening. (**C**) CT9 peptide interaction with the CL brings HCs into the “available to open” state. (**D**) Bell-shaped [Ca^2+^]_i_ dependency of HC opening. CT9 removes HC inhibition at above 500 nM [Ca^2+^]_i_ (dashed line), thereby strongly enhancing HC opening. (**E**) Caffeine activation of RyRs triggers [Ca^2+^]_i_ elevation (brown trace) followed by NCX current (black) associated with Ca^2+^ extrusion. Spiking HC opening activity appears superimposed on the NCX current. At the start of caffeine application, simultaneous (stacked spikes) HC openings can be distinguished resulting from up to three HCs. (**F**) HC opening in ventricular cardiomyocytes requires three conditions: (a) activation of RyR2 by caffeine, adrenergic stress, or rapid electrical pacing; (b) [Ca^2+^]_i_ elevation; and (c) molecular interaction between RyR2 and Cx43 HCs (inhibited by RyRHCIp peptide). (**G**) Open HCs carry inward current producing about 1.6 mV depolarization per HC (at –70 mV diastolic potential). HC Ca^2+^ entry triggers microdomain Ca^2+^ elevation that is extruded by NCX activity, adding another 1.3 mV depolarization, yielding almost 3 mV depolarization per HC. HC opening events are brief (~8 milliseconds on average) but sufficiently long to obtain steady-state membrane charge redistribution and achieve the approximately 3 mV depolarization estimate.

In addition to the interactions discussed above, the Cx43 CT tail also interacts with the CL-located L2/Gap19 region (Figure 1, interaction 3). In particular, interaction of the CT9 region with the L2/Gap19 region is necessary to bring Cx43 HCs to the “available to open” state (68, 69) (Figure 2A), from which HC opening can be triggered by electrical stimulation or elevation of the cytoplasmic Ca^2+^ concentration ([Ca^2+^]_i_). The [Ca^2+^]_i_-dependency of HC opening in ventricular cardiomyocytes is characterized by a bell-shaped relation (70) (Figure 2D) whereby Cx43 HCs open in the 100- to 500-nM [Ca^2+^]_i_ range but close again upon further [Ca^2+^]_i_ elevation to 500–1,000 nM. HC opening involves Ca^2+^/calmodulin (CaM) signaling and interaction with a CL-located CaM binding region (71, 72) (Figure 1). The loss of the response above 500 nM relates to CT linkage to the actomyosin cytoskeleton that disrupts CT-CL interaction (reviewed in refs. 73, 74). The bell-shaped [Ca^2+^]_i_-dependency of Cx43 HC opening is also observed in Cx43-expressing noncardiac cells (71, 75, 76). An important difference is that ventricular cardiomyocytes require two factors to trigger HC opening at negative diastolic membrane potential: a [Ca^2+^]_i_ elevation combined with simultaneous activation of the ryanodine receptor (RyR) by, e.g., caffeine or 4-chloro-*m*-cresol (70, 77). Note that these requirements are not necessary for electrical activation of Cx43 HC opening triggered by stepping of the membrane potential (V_m_) to positive voltages (70, 75).

In addition to the CT9 tail interaction with the CL-located L2/Gap19 region, a second CT-located SH3-binding domain (Figure 1, interaction 4) is also able to interact with the L2/Gap19 region and switch HCs to the “available to open” state (69).

Lowering of extracellular Ca^2+^ below approximately 0.5 mM is also a well-established trigger for HC opening, which occurs as a result of diminished Ca^2+^ interactions with negatively charged residues at the outer pore side of the HC (78). In principle, HC opening triggered by low extracellular Ca^2+^ should be independent of CT-CL interactions. However, low extracellular Ca^2+^ conditions may activate [Ca^2+^]_i_ dynamics, thereby recruiting tail-loop–dependent HC opening from the intracellular side (79).

### L2 and Gap19 prevent Cx43 tail-loop interactions.

Investigations on CT-CL control of Cx43 HC opening led to the identification of the Cx43 HC peptide inhibitors L2 and Gap19 (reviewed in refs. 22, 80) (Figure 1). L2 peptide was initially developed with the aim of preventing GJ closure upon intracellular acidification (see next paragraph) but was further scrutinized and found to act as a Cx43 HC inhibitor in various Cx43-expressing cells (68). Gap19 (19) is located within the L2 region, with residues 130–136 being part of a sequence that is crucial for interaction with the CT (81–84). Its CT binding was found to be stronger than that of L2 peptide, and it demonstrated pharmacological properties shown in Table 2. Gap19 brings Cx43 HCs into the “unavailable to open” state (Figure 2B). The peptide does not inhibit GJs upon brief exposure (hours) but enhances GJ coupling upon 24- to 48-hour incubation (~50% increase after 48 hours) (19). Gap19 is not a pore blocker but a gating modifier that reduces open-state gating (~220 pS) while increasing substate gating (~80 pS) (85), restricting the permeation to low-MW substances.

### L2 peptide prevents GJ closure.

L2 peptide was initially developed with the aim of preventing closure of Cx43-based GJs upon intracellular acidification, as occurs in cardiac ischemia (86, 87). The finding that L2 prevents GJ closure as well as HC opening has stimulated the search for other peptides fulfilling this specific purpose (88). For example, the octapeptide RRNYRRNY prevents GJ closure and inhibits plasmalemmal as well as mitochondrial HC opening (89). The insight that HC assembly into a GJ channel completely alters the functional outcome of tail-loop interactions has led to the understanding that the two channel types are distinctly regulated, often in opposite ways. As such, interfering with CT-CL interaction has the dual potential of preventing both the closure of Cx43 GJs and the opening of HCs, which offers an interesting opportunity to follow up on the disappointing outcome of the danegaptide study on myocardial infarction patients (45) with new candidate molecules based on RRNYRRNY and CyRP-71 “RXP”-type Cx43-binding peptides (88).

### CT9 enhances HC function and stimulates GJ incorporation of HCs.

In contrast to Gap19/L2, CT9 peptide enhances Cx43 HC function (19, 68, 90), and increases spiking HC opening activity in ventricular cardiomyocytes from mice (Figure 2, C and D) and pigs (77). The increased HC function results from two effects: first, CT9 peptide substitutes for the endogenous CT9 sequence, thereby maintaining HCs in the “available to open” state (Figure 2C). Second, CT9 peptide removes HC inhibition at above 500 nM [Ca^2+^]_i_, thereby strongly enhancing HC opening activity (68, 76) (Figure 2D).

Additionally, as delineated in the previous section, CT9 peptide (αCT1/αCT11) competes with the endogenous CT sequence for access to PDZ2/ZO-1, thereby stimulating HC incorporation into GJ plaques and reducing the lateralized HC pool and its associated channel opening activity. Increased channel gating obviously occurs faster than the process of HC incorporation into GJs, separating these two distinct effects along the timeline, resulting in acutely enhanced HC function followed by a slower phase of HC recruitment toward GJs associated with decreased HC activity and improved GJ coupling.

### Combining HC inhibition with stimulation of HC incorporation into GJs.

Can inhibition of HC opening and prevention of GJ closure be combined with enhancement of HC incorporation into GJs? In principle, combined treatment with Gap19 and CT9-based αCT1/αCT11 (to inhibit HC function as well as reduce HC presence in lateral membranes) looks like an interesting option for further exploration. However, in vivo data in mice demonstrated that such coadministration results in the mutual canceling of each peptide’s effect (91), either as a consequence of direct Gap19-CT9 interaction before they reach their respective targets on the Cx43 protein (68), or by the balancing of their functional effects on HCs. Alternatively, sequential administration of Gap19 to acutely inhibit HC opening followed by delayed intervention with CT9-based tools to enhance HC incorporation into GJs may be an option. However, such a phased approach would come with the risk of a temporary CT9-induced increase in HC activity and associated arrhythmic consequences (discussed in the next section). Further work is necessary to develop tools that stimulate HC insertion into GJs without first activating HC opening. Given the multitude of interactions of Cx43, either within a single Cx43 subunit, between subunits, or with other proteins, the field is open for further discovery.

### Tubulin and actin control HC trafficking and function.

The Cx43 CT contains a tubulin-binding motif that is responsible for direct interaction with microtubules (92, 93) (Figure 1), mediating forward delivery of Cx43-containing vesicles to the perinexus (4, 7, 94, 95) steered by the microtubule plus-end tracking protein EB1 (96). Upon arrival in the subsarcolemmal zone, Cx43 colocalizes with non-sarcomeric β-actin via intermediate accessory proteins such as ZO-1 and/or drebrin (97). Both proteins are likely candidates to function as intermediates between actin and Cx43 and may be involved in disrupting CT-CL interaction and HC closure at high [Ca^2+^]_i_ (Figure 2D). The Cx43 CT-tubulin interaction is prevented by phosphorylation of Tyr247 by Src/Tyk2, which is modulated in a CaM-dependent manner (98–100).

### Cx43 interacts with RyRs and controls HC opening.

A recent study using super-resolution microscopy revealed that, in mouse left ventricular cardiomyocytes, about half of the dyadic RyR2s colocalize within about 20 nm of Cx43 (77). Not only RyR2 but also sarcolemmal Ca_v_1.2 and Na^+^/Ca^2+^ exchange (NCX) transporter protein colocalized with Cx43, with 30% to 50% positioned within 20 nm distance from Cx43 (77). Importantly, these colocalization data were obtained from the cell end/peri-ID region where Cx43 is abundantly present, and not from other regions or the cell interior. These findings place Cx43 in direct proximity to the major players involved in [Ca^2+^]_i_ regulation and excitation-contraction coupling in the heart. The colocalization between Cx43 and RyR2 was pinned down to an interaction between a short aspartate-rich sequence on the CT tail that includes part of the α-helical H2 region on the Cx43 CT tail (70) (Figure 1, RyR2). RyR activation is associated with major conformational changes of the P1 domain (101–103), which is hypothesized to switch the HC to the “available to open” state. A peptide dubbed RyRHCIp based on a conserved sequence on the P1 domain of the RyR was found to inhibit Cx43 HC opening in ventricular cardiomyocytes triggered by RyR2 activation with caffeine (70), while having no effect in noncardiac cells that lack the high degree of differentiation. Interestingly, modification of the second Arg in the RyRHCIp stretch of the P1 domain (Arg1027) is phenotypically associated with primary familial hypertrophic cardiomyopathy (70).

### Cx43 allocates Na^+^ and K^+^ channels at IDs.

At the perinexus, Cx43 HCs interact with Na_v_1.5 (SCN5A), the pore-forming subunit of the voltage-gated Na^+^ channel that is essential for cardiomyocyte depolarization, and Kir2.1 K^+^ channels, associated with Andersen-Tawil syndrome and short QT syndrome (104–106). Two distinct pools of Na_v_1.5 channels exist in the heart: one located at the ID and one at the costameres (107, 108). As a result of these Cx43–Na^+^ channel interactions, Na^+^ currents are larger at the ID (108) and, most importantly, reduced Cx43 expression is associated with diminished Na_v_1.5 expression (109), thereby compromising the two main pillars of impulse conduction.

## Connexin HCs and arrhythmia

Due to the crucial role of Cx43 in the body and the heart in particular, mutants have a high risk of being eliminated at an early stage of fetal development (110–112), and thus, only a few reports link Cx43 mutations to human sudden cardiac death. Van Norstrand et al. identified two missense mutations in *GJA1* resulting in E42K and S272P mutations in Cx43 in sudden infant death syndrome (113). Additionally, Cx43 mutations have been identified as the cause of oculodentodigital dysplasia, an autosomal dominant syndrome characterized by craniofacial and limb abnormalities (114). Arrhythmias are strikingly absent in this disease except for the I130T mutant (located in the Gap19 sequence; Figure 1) that forms leaky HCs and is linked to ventricular tachycardia (17). More recently, several studies demonstrated that increased HC opening is linked to ventricular arrhythmogenesis at the cellular level, with evidence coming from mouse models of Duchenne muscular dystrophy (115–118) and of arrhythmogenic right ventricular cardiomyopathy (119), as well from human data on Cx43 HC-linked excitability disturbances leading to triggered APs (tAPs) in heart failure (77). These experimental studies will be further discussed below.

Atrial cardiomyocytes express both Cx40 and Cx43, with Cx40 showing the largest expression. Several mutations as well as a rare polymorphism have been reported in the Cx40 *GJA5* gene in human atrial fibrillation (AF) patients (120, 121), but little or nothing is known about their functional impact on Cx40-based HCs. As this Review focuses on Cx43 HCs, we include in the discussion below a recent report of Cx43 HC involvement in AF associated with a rare human mutation in the *MYL4* gene (c.234delC) as a heritable cause of AF (122).

### Duchenne muscular dystrophy.

**Figure 3:**
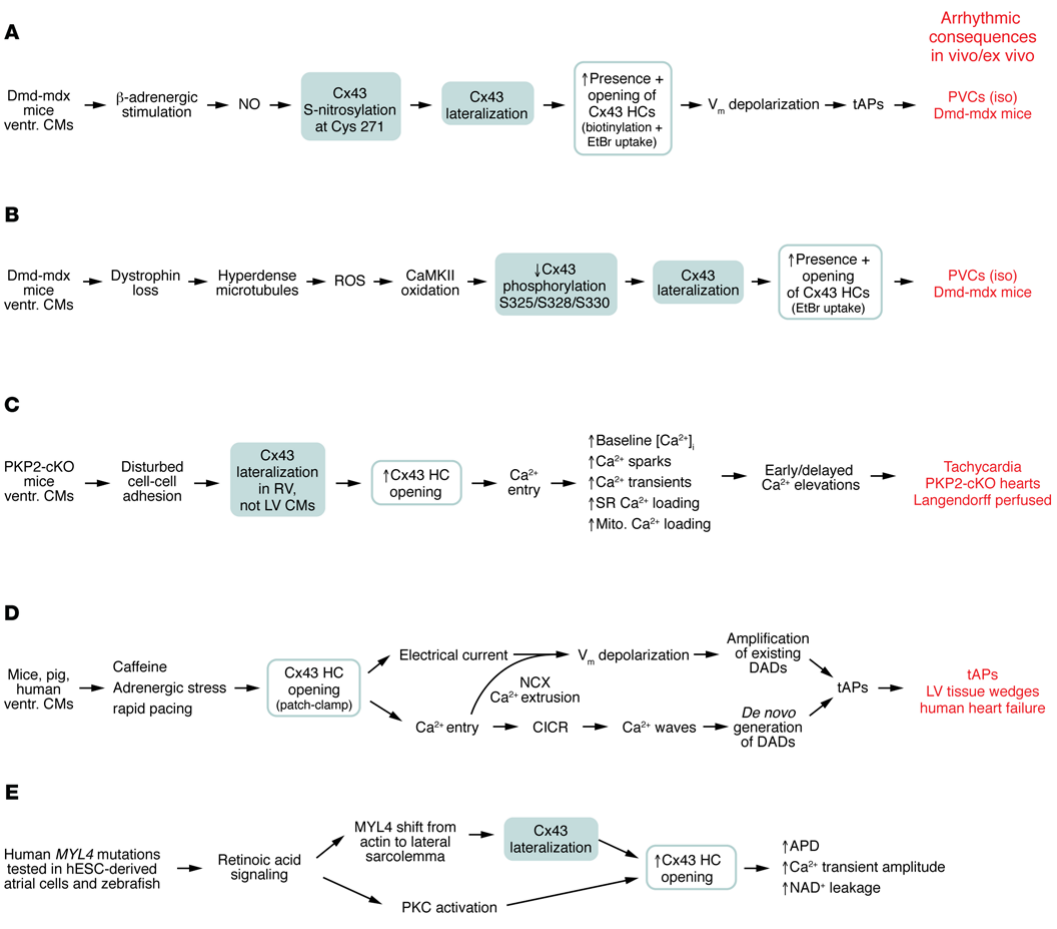
Contribution of Cx43 HCs to ventricular and atrial arrhythmogenesis. Overview of signaling cascades leading to HC-related arrhythmic responses in ventricular (**A**–**D**) and atrial cardiomyocytes (**E**). Arrhythmogenic consequences of HC opening are given for the ventricular cascades **A**–**E** and are further explained in the main text. APD, action potential duration; cKO, conditional knockout; CM, cardiomyocyte; NAD^+^, nicotinamide adenine dinucleotide; ventr., ventricular.

Duchenne muscular dystrophy (DMD) is an X-linked genetic disease resulting in muscle degeneration owing to the absence of the protein dystrophin in affected males; it also leads to dystrophic cardiomyopathy, which is a major cause of patient mortality (123). In mdx mice, a genetic DMD model expressing non-functional dystrophin (further referred to as Dmd-mdx), Gonzalez et al. demonstrated that arrhythmias induced by β-adrenergic stimulation with isoproterenol, as well as the ensuing animal mortality, were significantly decreased after HC inhibition with Gap26 or Gap19 (115). ECG recordings demonstrated increased premature ventricular contractions (PVCs) in Dmd-mdx relative to wild-type animals (Figure 3A). The increased PVC frequency resulted from increased HC opening, based on ethidium bromide (EtBr) dye uptake studies. Increased HC opening resulted in depolarization of ventricular cardiomyocytes, leading to “triggered activity,” i.e., above-threshold depolarization events manifested as tAPs (117), a cellular arrhythmic manifestation. Increased HC opening resulted from isoproterenol-induced nitric oxide formation, leading to nitrosylation of Cx43 at Cys271 (Figure 1) and subsequent lateralization of Cx43 (Figure 3A). Accordingly, biotinylation experiments showed an increased density of lateralized HCs (117).

In a further scrutiny of Dmd-mdx mice, Himelman et al. reported that dystrophin loss is associated with microtubule reorganization as hyperdense structures (118), which were found to enhance ROS production and oxidation of calmodulin kinase II (CaMKII) (Figure 3B). Oxidized CaMKII consequently provoked a decrease in Cx43 phosphorylation of Cx43 residues S325/S328/S330 (Figure 1) that set the stage for Cx43 remodeling, lateralization, and increased HC opening. Accordingly, the isoproterenol-triggered PVC frequency correlated well with EtBr HC dye uptake. Moreover, PVCs were absent in phosphomimetic Cx43 S325E/S328E/S330E mice, which also displayed decreased cardiomyopathy and increased survival. The QT time, reflecting the AP plateau phase and repolarization, was prolonged in Dmd-mdx mice while normal in S325E/S328E/S330E animals. The observed beneficial effect of phosphomimetic S325E/S328E/S330E is in line with the observation that phosphorylation of the Ser triplet is associated with proper localization of Cx43 to the IDs, while hypophosphorylation leads to lateralization (124–126) and increased probability of HC opening. In contrast, transgenic animals carrying a slightly different non-phosphorylatable mutant S325A/S328T/S330A showed decreased, not increased, HC activity as investigated by patch clamp approaches in mouse ventricular cardiomyocytes (127), which requires further scrutiny.

### Arrhythmogenic right ventricular cardiomyopathy.

Strict spatiotemporal control of [Ca^2+^]_i_ dynamics is crucial for normal cardiomyocyte function, and recent evidence demonstrated that Cx43 HC opening triggers disturbed [Ca^2+^]_i_ dynamics in a plakophilin-2–knockout (PKP2-KO) mouse model of arrhythmogenic right ventricular cardiomyopathy (ARVC) (119). PKP2 is a desmosomal ID protein involved in regulating intercellular junction assembly (128); it also controls gene transcription related to Ca^2+^ cycling and heart rhythm (129). Mutations in *PKP2* are associated with the majority of genetic causes leading to ARVC (130). Ventricular cardiomyocytes isolated from inducible-PKP2-KO (PKP2-cKO) mice before the appearance of overt cardiomyopathy (i.e., still-intact tissue) have a widened intercellular space and display Cx43 remodeling and HC lateralization in the right but not the left ventricle (119) (Figure 3C). Interestingly, the phenotype is associated with ultrastructural appearance of linear plaque-like HC arrays (119, 129, 131). At the functional level, the phenotype demonstrated abnormal [Ca^2+^]_i_ dynamics that were diminished in PKP2-cKO/Cx43^+/–^ mice and suppressed by Gap19. The disturbed [Ca^2+^]_i_ dynamics entailed increased sparking frequency and Ca^2+^ transient amplitudes, as well as increased sarcoplasmic reticulum (SR) and mitochondrial Ca^2+^ loading, culminating in early and delayed Ca^2+^ alterations as cellular arrhythmogenic signs. In addition to Gap19 inhibition of HCs, the increased Ca^2+^ spark frequency was also reduced by PKC inhibitors. PKP2-cKO cardiomyocytes further displayed increased phosphorylation of RyR2 at amino acid residue T2809 located in a domain involved in modulating Ca^2+^ gating. These cellular data were complemented by ECG studies in Langendorff-perfused hearts of PKP2-cKO mice, which, in contrast to wild-type animals, demonstrated long runs of ventricular tachycardia upon challenge with isoproterenol/rapid pacing (Figure 3C).

### HC-linked arrhythmogenic mechanisms in ventricular cardiomyocytes.

De Smet et al. and Lissoni et al. provided a detailed account of the mechanisms and consequences of Cx43 HC opening in response to challenge of mouse and pig left ventricular cardiomyocytes with caffeine or β-adrenergic agonists (70, 77). A typical caffeine response is represented in Figure 2E, demonstrating a slow transient inward current on which, superimposed, appear brief spike-like current events. The transient current is mediated by the electrogenic action of the Na^+^/Ca^2+^ exchange (NCX) transporter, which for every Ca^2+^ extruded brings three Na^+^ inside the cell (Figure 2G). In contrast, the spiking currents result from brief Cx43 HC opening events (first linked to Cx43 in 1990 by Pott and Mechmann, ref. 132). Other proposed channels, like sarcolemmal RyRs (133, 134), Panx1 channels (135), and the transient receptor potential channels TRPP2 (PKD2) and TRPP5 (PKD2L2) (136), were excluded (77). Atrial cardiomyocytes display similar spiking opening activity of Cx43 HCs (137). In ventricular cells, HC activity was found to vary between species, with mouse and human cardiomyocytes displaying the highest activity while activity in pig cells was lower (77). It is often assumed that the high HC opening activity in single dissociated cardiomyocytes is a by-product of cell dissociation or linked to the absence of GJs. However, macro-patch approaches mapping HC activity at cell ends showed that HC opening activity was larger in GJ-connected cardiomyocyte cell pairs than in single cardiomyocytes (77).

HC opening in ventricular cardiomyocytes at negative diastolic potential requires both caffeine stimulation and [Ca^2+^]_i_ elevation; importantly, [Ca^2+^]_i_ elevation by itself is not sufficient and neither is caffeine stimulation under conditions of strong [Ca^2+^]_i_ buffering (70). It was found that Cx43 interacts with RyR2, and preventing this interaction using RyRHCIp peptide blocked HC activation. These findings suggest three prerequisites to open Cx43 HCs in ventricular cardiomyocytes: (a) activation of RyR2 by caffeine, adrenergic stress, or rapid pacing; (b) [Ca^2+^]_i_ elevation; and (c) molecular interaction between RyR2 and Cx43 (Figure 2F).

Cx43 HC opening in ventricular cardiomyocytes can also be triggered by isoproterenol, and combined isoproterenol/rapid pacing stimulation results in very strong HC opening activity (77). These stimulation protocols are well known to increase SR Ca^2+^ loading, leading to increased Ca^2+^ sparking activity (138–140). However, Ca^2+^ release combined with RyR2-Cx43 interaction fulfills only two, not all three, of the conditions necessary for HC activation, but it seems obvious that the third condition of RyR2 activation is mediated by Ca^2+^ itself, as occurs in Ca^2+^-induced Ca^2+^ release (CICR).

How does HC opening lead to arrhythmogenic responses in ventricular cardiomyocytes? De Smet et al. demonstrated that HC opening frequently occurs in association with Ca^2+^ waves triggered by β-adrenergic activation combined with rapid pacing (77). Such Ca^2+^ waves typically lead to delayed afterdepolarizations (DADs), caused by the electrogenic consequences of NCX-mediated Ca^2+^ extrusion during the wave (entry of three Na^+^ for every Ca^2+^ extruded). HC opening results in an approximately 1.6-mV depolarization per open HC and another of about 1.3 mV resulting from electrogenic effects of NCX extrusion of HC-linked elevated microdomain [Ca^2+^]_i_ (77) (Figure 2G and Figure 3D). As such, HC spiking activity occurring during a Ca^2+^ wave may thus increase DAD amplitude and tAP frequency. This was confirmed in human ventricular cardiomyocytes from failing hearts, where HC inhibition with TAT-Gap19 significantly decreased DAD amplitude and tAP frequency.

In addition to HC activity occurring during Ca^2+^ waves, HC opening was also found to precede the Ca^2+^ waves. In this case, there was a brief approximately 50-millisecond delay between HC opening activity and the start of the Ca^2+^ wave, suggesting a causal linkage (77). The majority of such waves started at cell ends where HC density is highest. Opening of HCs at these sites not only triggers depolarization but also leads to Ca^2+^ entry and [Ca^2+^]_i_ elevation at HC-dyad microdomains estimated to attain 0.8 μM per HC for 1.0 mM extracellular Ca^2+^ (3.4 μM for 1.8 mM external Ca^2+^) (77). Such HC-initiated Ca^2+^ waves invariably led to DADs and tAPs in ventricular cardiomyocytes from failing human hearts (see *De novo generation of DADs* in Figure 3D), which were effectively suppressed by TAT-Gap19. These cellular data were confirmed in arterially perfused ventricular tissue wedges from failing human hearts demonstrating significantly increased DAD rates (~1 per 3 seconds) and tAPs (~1 per 7 seconds) following 2 Hz pacing/isoproterenol, compared with wedges from non-failing hearts (Figure 3D). DAD amplitudes and DAD/tAP frequencies were all significantly reduced by TAT-Gap19 and increased by TAT-CT9.

Collectively, these observations indicate that the opening of a few Cx43 HCs per cardiomyocyte (sometimes one to four coincident HC openings; Figure 2E) may be sufficient to produce arrhythmogenic responses in ventricular cells and tissues from failing human hearts. It is important to note here that HC opening will only result in depolarization when opening occurs at negative V_m_, i.e., during diastole. HCs are poorly selective channels with a reversal potential of around 0 mV. Consequently, HC opening at positive V_m_, e.g., during the AP plateau or early repolarization, will result in V_m_ changes in the direction of the 0-mV axis, i.e., opposite to the responses at negative V_m_.

Figure 3D summarizes the signaling cascade starting from stressors (caffeine, isoproterenol, tachycardia) leading to increased HC opening in ventricular cardiomyocytes and its arrhythmic consequences in heart failure. Based on the evidence presented in the cascades of Figure 3, A–D, we conclude that arrhythmic HC contributions may occur through three distinct mechanisms summarized in Table 3 above.

### Cx43 HCs in AF.

While most evidence for arrhythmogenic contributions of HCs comes from work on ventricular cells and tissues, some evidence also points to potential arrhythmogenic involvement of HCs in the atria. Ghazizadeh et al. (141) used an original approach starting from a rare human mutation in the *MYL4* gene (Icelandic c.234delC), coding for myosin light chain-4 (MYL4) protein and representing one of the identified heritable causes of AF (122). They explored mutant *MYL4* properties in atrial cells derived from human embryonic stem cells (hESCs) and in zebrafish\, in which the *cmlc1* gene is a putative ortholog of human *MYL4*. To include more common AF-generating mechanisms, they applied database information from atrial patient biopsies and tested specific *MYL4* mutations at the single-cell (hESC) and organ (zebrafish) levels to explore structural, functional, and transcriptional features predisposing to AF. The work pointed to involvement of retinoic acid signaling in cardiomyocyte cell polarity in mutant *MYL4* (Figure 3E) and demonstrated that MYL4 associated with F-actin in atrial biopsies from human subjects in normal sinus rhythm, while MYL4 shifted to the sarcolemma in biopsies from AF patients. MYL4 also coimmunoprecipitated with Cx43, and Cx43 association with actin was decreased in MYL4^–/–^ hESCs, which we suggest removes the high [Ca^2+^]_i_ brake on HC activity (Figure 2D) and thus leads to increased HC opening. In terms of electrical and Ca^2+^ consequences, experiments in MYL4^–/–^ zebrafish demonstrated increased AP duration (APD) and Ca^2+^ transient amplitudes in comparison with MYL4^+/+^ animals. Interestingly, NAD^+^, a compound known to be released through Cx43 HCs (142–144), was also elevated. These MYL4^–/–^-associated alterations in APD, Ca^2+^ transients, and NAD^+^ release were all suppressed by Gap19 inhibition of Cx43 HCs. It was furthermore shown that retinoic acid signaling enhanced PKC activity and inhibition of PKC mitigated the changes in APD, Ca^2+^ transients, and NAD^+^ release. Taken together, this study brings up three reasons for increased HC function in atrial cells: (a) Cx43 lateralization, (b) PKC activation, and (c) actomyosin-linked disturbances favoring HC opening when [Ca^2+^]_i_ rises above 500 nM, as suggested here.

The multitude of Cx43 interactions with other proteins makes “location” of Cx43 a crucial determinant of its channel functions. In this regard, cardiomyocytes derived from induced pluripotent stem cells still lack the extreme degree of cardiomyocyte differentiation, in particular at the level of T-tubules, CICR, and the IDs where HCs reside in perinexal areas surrounding the GJ plaques (4, 58, 104–106, 145–149). Thus, conclusions regarding altered HC presence and function always need to be verified in experiments on primary cardiomyocytes.

## Conclusions

Our understanding of the factors that control Cx43 HC function and their contribution to ischemic heart disease and ventricular tachyarrhythmias is rapidly growing. Several peptide tools have become available to selectively inhibit HC opening as well as to enhance HC incorporation into GJs, which has substantially increased our comprehension of HC roles in disturbed cardiomyocyte function and basic cellular arrhythmogenic mechanisms. It must be considered, though, that despite the broad clinical use of beta blockers mitigating key molecular targets of cardiac excitability and despite clinical trials focused exclusively on GJs, none of these treatments has demonstrated a mortality benefit (45, 150, 151). As such, there is room for thinking about novel multilevel approaches aimed at preserving Cx43 trafficking, preventing HC opening and GJ closure, and driving HCs toward integration into GJ plaques that keep the syncytium connected. Thus, the stage is set to develop current insights toward novel candidate pharmacological tools for experimental as well as therapeutic application in human patients.

## Figures and Tables

**Table 3:**
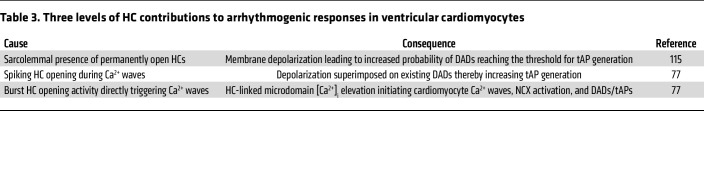
Three levels of HC contributions to arrhythmogenic responses in ventricular cardiomyocytes

**Table 2:**
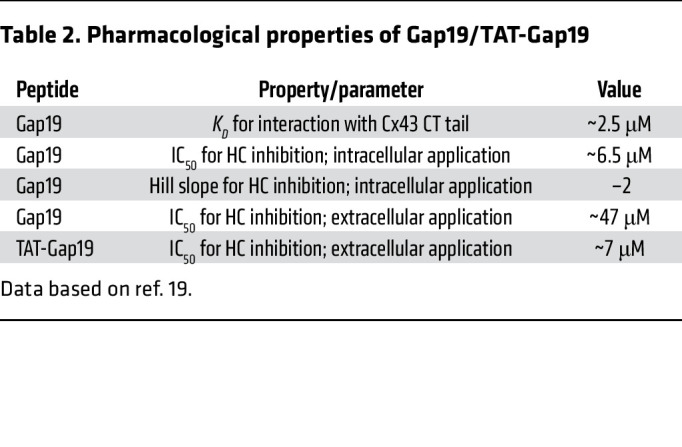
Pharmacological properties of Gap19/TAT-Gap19

**Table 1:**
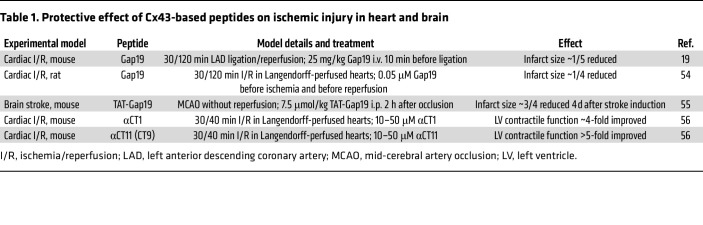
Protective effect of Cx43-based peptides on ischemic injury in heart and brain

## Version 1 03/15/2023

Electronic publication
